# Comparative Analysis of Growth, Survival, and Virulence Characteristics of *Listeria monocytogenes* Isolated from Imported Meat

**DOI:** 10.3390/microorganisms12020345

**Published:** 2024-02-07

**Authors:** Xinye Pan, Jinling Shen, Yi Hong, Yufan Wu, Dehua Guo, Lina Zhao, Xiangfeng Bu, Leijie Ben, Xiang Wang

**Affiliations:** 1 School of Health Science and Engineering, University of Shanghai for Science and Technology, Shanghai 200093, China; pxy1222202@163.com (X.P.); ussthy@163.com (Y.H.); bxiangfeng@163.com (X.B.); benleijie123@163.com (L.B.); 2 Technology Center for Animal Plant and Food Inspection and Quarantine of Shanghai Customs, Shanghai 200135, China; jinling_zhan19@163.com (J.S.); guodehua@customs.gov.cn (D.G.); lhfzln@126.com (L.Z.); 3 Centre of Analysis and Test, School of Chemistry & Molecular Engineering, East China University of Science and Technology, Shanghai 200237, China; wuyufan@ecust.edu.cn

**Keywords:** foodborne pathogen, variability, biological characteristics, virulence, potential pathogenicity

## Abstract

*Listeria monocytogenes* is an important foodborne pathogen with worldwide prevalence. Understanding the variability in the potential pathogenicity among strains of different subtypes is crucial for risk assessment. In this study, the growth, survival, and virulence characteristics of 16 *L. monocytogenes* strains isolated from imported meat in China (2018–2020) were investigated. The maximum specific growth rate (*μ_max_*) and lag phase (*λ*) were evaluated using the time-to-detection (TTD) method and the Baranyi model at different temperatures (25, 30, and 37 °C). Survival characteristics were determined by *D*-values and population reduction after exposure to heat (60, 62.5, and 65 °C) and acid (HCl, pH = 2.5, 3.5, and 4.5). The potential virulence was evaluated via adhesion and invasion to Caco-2 cells, motility, and lethality to *Galleria mellonella*. The potential pathogenicity was compared among strains of different lineages and subtypes. The results indicate that the lineage I strains exhibited a higher growth rate than the lineage II strains at three growth temperatures, particularly serotype 4b within lineage I. At all temperatures tested, serotypes 1/2a and 1/2b consistently demonstrated higher heat resistance than the other subtypes. No significant differences in the log reduction were observed between the lineage I and lineage II strains at pH 2.5, 3.5, and 4.5. However, the serotype 1/2c strains exhibited significantly low acid resistance at pH 2.5. In terms of virulence, the lineage I strains outperformed the lineage II strains. The invasion rate to Caco-2 cells and lethality to *G. mellonella* exhibited by the serotype 4b strains were higher than those observed in the other serotypes. This study provides meaningful insights into the growth, survival, and virulence of *L. monocytogenes*, offering valuable information for understanding the correlation between the pathogenicity and subtypes of *L. monocytogenes*.

## 1. Introduction

*Listeria monocytogenes* is a Gram-positive foodborne pathogen that occasionally causes listeriosis, a severe illness that can be fatal in people with low immunity, including the elderly (aged 65 years or older) and pregnant women and their neonates [1]. The rapid dissemination of *L. monocytogenes* is facilitated by the intricate network of global trade and transportation. The prevalence of *L. monocytogenes* in imported food, coupled with outbreaks of associated foodborne illnesses, has been reported worldwide. In 2016 and 2020, outbreaks of *L. monocytogenes* infection caused by enoki mushrooms imported from the Republic of Korea occurred in the United States and Canada, which included 48 patients [2]. In 2011, an outbreak of nine listeriosis cases in Switzerland was linked to the consumption of imported cooked ham [3]. Intact packages of imported ricotta salata led to a *L. monocytogenes* outbreak, resulting in 22 cases across 14 jurisdictions of the U.S. in 2012 [4]. A previous study identified 57 *L. monocytogenes* isolates from 1474 illegally imported food samples into the European Union (EU), originating from 17 different countries [5]. In China, a study conducted from 2018 to 2020 revealed an overall prevalence of 5.62% for *L. monocytogenes* in 1797 imported food samples [6]. The spread of *L. monocytogenes* in imported food worldwide presents challenges in food safety, so it is necessary to study the various strains’ pathogenic potential in order to provide a scientific basis for related risk assessment.

The pathogenicity of *L. monocytogenes* depends on its growth characteristics, survival ability in adverse environments, and the virulence of the specific strain [7]. It may not be appropriate to assume uniform pathogenicity across all *L. monocytogenes* strains. Molecular epidemiological evidence suggests considerable variability in the distribution of *L. monocytogenes* in food production and food processing environments, as well as in human clinical listeriosis cases [8,9]. Serotypes 1/2a and 1/2c within lineage II are commonly identified in food and environmental samples [10,11]. The high incidence of recombination compared to point mutation in lineage I may contribute to the competition and survival of strains in diverse environments [12]. Globally, serotypes 1/2a, 1/2b, 1/2c, and 4b constitute the majority of human listeriosis cases [13,14,15,16]. Furthermore, many animal and in vitro studies indicate that lineage I isolates generally exhibit greater virulence than lineage II isolates [17,18]. Therefore, diverse characteristics, such as growth patterns, resistance to environmental stress, and virulence, are observed among different strains of *L. monocytogenes*. Recognizing these variations in pathogenic potential is essential for evaluating the associated risks when these strains are present in food products.

The objective of this study was to comprehensively analyze and compare the diverse characteristics of 16 *L. monocytogenes* strains previously isolated from imported meat [6].

We examined their survival characteristics (growth fitness and stress tolerance), virulence factors (adhesion and invasion to Caco-2 cells and motility), and their lethality in a model species (*Galleria mellonella*) of strains, which was expected to better evaluate the pathogenic risk of *L. monocytogenes* from imported food.

## 2. Materials and Methods

### 2.1. Strains and Culture Conditions

A total of 16 strains isolated from imported meat between 2018 and 2020 (Table 1) and 1 ATCC strain (ATCC19112) were used in this study [6]. Single-strain stock cultures were inoculated into tryptic soy broth containing 0.6% yeast extract (TSB-YE, Hopebio, Qingdao, China) and incubated for 24 h at 37 °C. The cultures were then plated on trypticase soy agar containing 0.6% yeast (TSA-YE, Hopebio, Qingdao, China), incubated for 24 h at 37 °C, and stored at −4 °C as working cultures. Prior to the experiments, an individual colony was picked and inoculated into TSB-YE and incubated at 37 °C for 24 h, yielding an initial bacterial suspension with a concentration of approximately 10^9^ CFU/mL.

### 2.2. Growth Characteristics

To evaluate the growth characteristics of *L. monocytogenes*, an automated growth curve analyzer (Bioscreen C, Lab Systems, Helsinki, Finland) was employed for real-time monitoring of the optical density (OD) values. The investigation focused on understanding the bacterial growth patterns at 25 °C, 30 °C, and 37 °C. The experimental approach followed the methodology outlined by Zhao et al. [19]. The determination of the maximum specific growth rate (*μ_max_*) was performed through time-to-detection (TTD) calculations, employing the formula:

$$\mu_{max}=\frac{-1}{K}$$

where *K* denotes the slope derived from the linear regression analysis of the logarithmic values of bacterial concentrations at different dilutions against the corresponding detection times.

Using the obtained *μ_max_* values, the Baranyi model [20] was employed to calculate the lag phase (*λ*). The calculation is represented by the formula:

$$\lambda =T_{d}-\frac{\ln N_{d}-\ln N_{0}}{\mu_{max}}$$

where *T_d_* signifies the time (in hours) required for the initial bacterial concentration (OD value reaching 10^7^ CFU/mL), *N_d_* represents the colony concentration (CFU/mL) at the detection time, and *N*_0_ is the initial bacterial concentration (CFU/mL). 

### 2.3. Heat Resistance

The heat resistance of *L. monocytogenes* was characterized using the apparent *D*-value, following the methodology outlined in a previous study by Xiang et al. (2021) [21]. The bacterial suspension was subjected to continuous ten-fold dilutions to determine the initial bacterial concentration. Heat treatments were carried out in a PCR instrument (Analytik Jena AG Co., Ltd., Jena, Germany), 30 μL of stationary phase cells were transferred to a 0.1 mL fast reaction tube with cap (KINRGEN, Shanghai, China) and heated according to the specified program. The temperature thermal treatments were as follows: 60 °C for 3–5 min, 62.5 °C for 1–2 min, and 65 °C for 0.5–1 min. Following the completion of the heat inactivation process, the tubes were immediately transferred to an ice-water bath to stop further heat deactivation of the cells. Post-cooling, 20 μL of the bacterial suspension was extracted and diluted ten-fold in 180 μL of sterile physiological saline and further enumerated on TSAYE plates. The formula for calculating the apparent *D*-value is expressed as:

$$D=\frac{t}{Log\left(N_{0}\right)-Log(N_{t})}$$

where *t* is the heat treatment time (minutes), *N*_0_ is the initial bacterial concentration (CFU/mL), and *N_t_* is the bacterial concentration after heat treatment (CFU/mL).

### 2.4. Acid Resistance

The acid resistance of *L. monocytogenes* was assessed by comparing the reduction in the cell count after treatment with acidified TSB-YE media (acidified with HCL) at pH = 2.5, 3.5, and 4.5. The initial bacterial suspension underwent continuous ten-fold dilutions to determine the initial bacterial concentration. To conduct the acid treatment, 0.5 mL of the bacterial suspension was mixed with 4.5 mL of acidified media and incubated at 4 °C for 21 h. After incubation, the mixture was centrifuged at 1405× *g* for 5 min and washed once with non-acidified TSBYE. The resulting cell pellet was suspended in 5 mL of sterile physiological saline for gradient dilution and cell counting. The log reduction in the *L. monocytogenes* count after the acid treatment was calculated as follows:

Log(*N_D_*) = Log(*N_i_*) − Log(*N_acid_*)

where *N_D_* is the reduction in the cell count (CFU/mL), *N_i_* is the initial bacterial count (CFU/mL), and *N_acid_* is the remaining bacterial count after the acid treatment (CFU/mL).

### 2.5. Caco-2 Cell Adhesion and Invasion Assay

The adherence and invasion assay was carried out using Caco-2 cells. In the cell culture process, Caco-2 cells were initially inoculated into a 12-well cell culture plate containing a customized medium [22] comprising 78% high-glucose DMEM, 20% fetal bovine serum, 1% penicillin–streptomycin solution, and 1% non-essential amino acids. The cells were then incubated at 37 °C with 5% CO_2_ in a constant-temperature incubator for a duration of 40 h. Subsequently, the culture medium was removed, the cells were washed with 1× PBS, and 1 mL of DMEM medium was added to each well.

For the bacterial preparation, each *L. monocytogenes* culture was centrifuged at 1405× *g* for 2.5 min to obtain a bacterial concentration of 10^9^ CFU/mL. After resuspending the pellet in 1 mL of DMEM, 10 μL of the bacterial suspension was added to each well, and the culture plate was gently tilted to ensure even distribution. Incubation was at 37 °C with 5% CO_2_ for 1.5 h, during which a drop plate count of the original bacterial suspension was performed to determine the actual number of infective bacteria. After the infection period, an adhesion assay was conducted by removing the liquid from each well, washing with 1× PBS, and adding 1 mL of 1% (*v*/*v*) Triton X solution. This was left for 3–5 min to facilitate cell detachment and rupture. The resulting cell lysate underwent gradient dilution, plating, and counting to determine the number of adherent bacteria. Following the infection, the invasion assay involved discarding the liquid, washing with 1× PBS, and adding 1 mL of cell culture medium containing dual antibiotics. Incubation at 37 °C with 5% CO_2_ for 1 h aimed to eliminate both adherent and free bacteria. After discarding the liquid and washing twice with 1× PBS, 1 mL of 1% (*v*/*v*) Triton X solution was added and left for 3–5 min. Similar to the adhesion assay, gradient dilution of the resulting cell lysate, plating, and counting were performed to determine the number of invasive bacteria calculated as follows:

$$P_{1}=\frac{N_{1}}{N_{0}}\times 100\%$$

$$P_{2}=\frac{N_{2}}{N_{0}}\times 100\%$$

where *P*_1_ is the adhesion rate (%), *P*_2_ is the invasion rate (%), *N*_1_ is the number of adherent bacteria (CFU/mL), *N*_2_ is the number of invasive bacteria (CFU/mL), and *N*_0_ is the actual number of infecting bacteria (CFU/mL).

### 2.6. Motility Assessment

The motility of *L. monocytogenes* was assessed following a modified protocol based on the methodology by Wu et al. [23]. Two types of semi-solid LB agar were prepared fresh before each experiment: 0.2% for the swimming assay and 0.3% for the swarming assay. The bacterial culture was adjusted to 10^7^ CFU/mL, and 2 μL were carefully inoculated onto the respective agar plates. In the swimming test, the suspension was pipetted onto the surface of the 0.2% agar, and the plates were incubated at 25 °C for 2 days. For the swarming test, a sterile needle was used to carefully stab the suspension into the 0.3% agar, followed by incubation at 25 °C for 4 days. After incubation, the motility zone formed by bacterial migration was measured (length and diameter), and the average values were calculated.

### 2.7. Galleria mellonella Infection Experiment

This experiment employed mature larvae of *Galleria mellonella* (Shanghai Payuan, Shanghai, China) (approximately 6 weeks old with a body length of around 2 cm). Bacterial cultures in the stationary phase were diluted to a concentration of 10^8^ CFU/mL using PBS buffer. Subsequently, 10 μL of the prepared bacterial suspension was injected into the hemocoel of each larva (at the location of the fourth pair of prolegs on the right side). Each bacterial strain was injected into 10 larvae, and the experiment was repeated three times. The negative control group received an injection of 10 μL PBS. The treated larvae were incubated in darkness at 37 °C, and their survival was monitored every 24 h until the fifth day [24]. Larvae that exhibited no response to external stimuli, such as body flipping or dish shaking, were considered dead. The time required to kill over 50% of the larvae (lethal time of 50%, LT50) was recorded.

### 2.8. Statistical Analysis

The growth, inactivation, and Caco-2 cell assay of each isolate were performed in two independent trials. Three independent trials were performed for the motility assessment and *G. mellonella* infection experiment of each isolate. Statistical analysis was performed using GraphPad Prism (Version 5.01, GraphPad Software, San Diego, CA, USA) and IBM SPSS (Version 25, IBM Corp., Armonk, NY, USA). Unpaired t-tests and analysis of variance (ANOVA) analysis were used to explore whether there were significant differences in the pathogenicity among strains, lineages, and serotypes (*p* < 0.05 was considered statistically significant).

## 3. Results

### 3.1. Growth Characteristics at Different Temperatures

**Figure 1:**
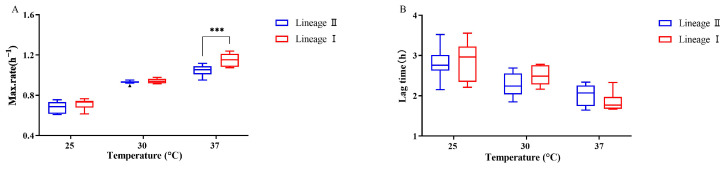
The *μ_max_* (**A**) and *λ* (**B**) of *L. monocytogenes* strains at 25 °C, 30 °C, and 37 °C based on lineage. The symbol (***) indicates significant difference in the representative data (*p* < 0.001).

**Figure 2:**
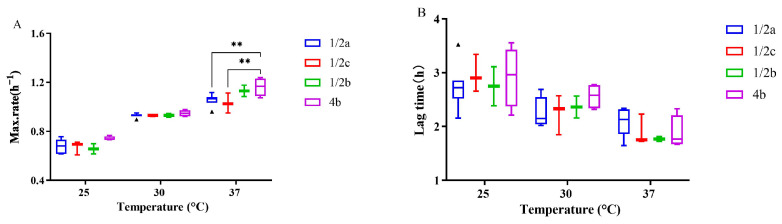
The *μ_max_* (**A**) and *λ* (**B**) of *L. monocytogenes* strains at 25 °C, 30 °C, and 37 °C based on serotype. The symbol (**) indicates significant difference in the representative data (*p* < 0.01); The symbol (▴) indicates moderated outliers.

The growth characteristics of pathogenic bacteria under favorable conditions play a crucial role in determining their proliferation in food. The time-to-detection (TTD) method was employed to quantitatively assess the growth characteristics of *L. monocytogenes* strains at different temperatures (25 °C, 30 °C, and 37 °C), and the obtained data were fitted to determine the maximum specific growth rate (*μ_max_*) and lag phase (*λ*) (Table S1). Across the three temperatures, the *μ_max_* of the *L. monocytogenes* strains ranged from 0.6 to 1.2 h^−1^, with a lag phase between 1.6 and 3.5 h. As the temperature increased, the *μ_max_* generally increased for each strain, while the *λ* showed a decreasing trend. The analysis revealed a highly significant impact of temperature on the *μ_max_* of both the lineage I and lineage II strains (*p* < 0.05) (Figure 1). Additionally, at 37 °C, the lineage I strains exhibited a significantly higher *μ_max_* compared to the lineage II strains (*p* < 0.05) (Figure 1). This indicates a potential temperature-dependent difference in the growth rate between these lineages. The growth parameters among different serotypes revealed that the serotype 4b strains consistently displayed the highest average *μ_max_* at all temperatures (Figure 2). Notably, at 37 °C, the serotype 4b strains had a significantly higher *μ_max_* compared to both the serotype 1/2a and 1/2c strains (*p* < 0.05).

### 3.2. Survival Characteristics after Heat Treatment

**Figure 3:**
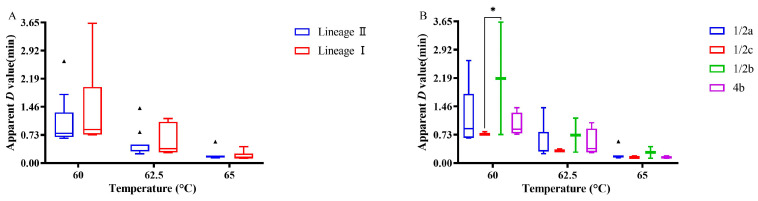
The *D*-value of *L. monocytogenes* strains based on lineage (**A**) and serogroup (**B**) at 60 °C, 62.5 °C, and 65 °C. The symbol (▴) indicates moderated outliers; error bars show standard errors. The symbol (*) indicates significant difference in the representative data (*p* < 0.05).

Heat treatment is one of the most crucial processing methods in food production and preservation. The heat tolerance of pathogenic bacteria can impact their survival levels in foods. The heat resistance comparison of the 16 strains at 60 °C, 62.5 °C, and 65 °C is illustrated in Figure 3. Thermal resistance of the 16 strains studied is illustrated in Figure 3. The apparent D-values at 60 °C, 62.5 °C, and 65 °C ranged from 0.65 to 3.63 min, 0.24 to 1.42 min, and 0.13 to 0.55 min, respectively. Notably, a highly significant difference in heat resistance was observed among the strains (*p* < 0.05) (Supplementary Figure S1). Specifically, strains L820 and L1393 displayed exceptionally high heat resistance compared to other strains (*p* < 0.05) (Supplementary Figure S1). The difference in heat resistance between the lineage I and lineage II strains was not significant (Figure 3A). At all temperatures tested, the serotype 1/2a and 1/2b strains exhibited higher apparent *D*-values than the other two subtypes (serotype 1/2c and serotype 4b), indicating greater heat tolerance. However, a statistically significant difference in the apparent *D*-values between the serotype 1/2a and 1/2b strains was observed only at 60 °C (Figure 3B).

### 3.3. Survival Characteristics after Acid Treatment

**Figure 4:**
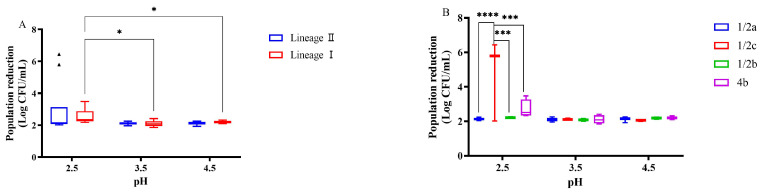
The population reduction of *L. monocytogenes* strains based on lineage (**A**) and serogroup (**B**) at pH 2.5, pH 3.5, and pH 4.5. The symbol (*), (***) and (****) indicates significant difference in the representative data (*p* < 0.05), (*p* < 0.001) and (*p* < 0.0001). The symbol (▴) indicates moderated outliers.

Acidic conditions are common in the food chain, and resistance to acidity reflects the survival capability of *L. monocytogenes*. The acid resistance of *L. monocytogenes* was assessed at pH 2.5, 3.5, and 4.5, analyzing bacterial reduction as a measure of acid susceptibility. The bacterial reduction after the acid treatment of *L. monocytogenes* is depicted in Figure 4. At the three pH values studied, the average log reductions were 2.78 (pH 2.5), 2.10 (pH 3.5), and 2.14 (pH 4.5). Notably, at pH 2.5, four strains displayed significantly higher reduction than the others (*p* < 0.05). In particular, strains L573 and L389 exhibited exceptional sensitivity to the acidic environment, with an average reduction exceeding 6 Log CFU/mL, much higher than the 2.02–3.47 Log CFU/mL range observed for other strains (Supplementary Figure S2). At pH 2.5, no significant difference in the log reduction was found between the lineage I and lineage II strains (Figure 4A). However, the acid treatment conditions significantly impacted the log reduction of the lineage I strains, with pH 2.5 leading to a significantly higher reduction than the other two acidic environments (*p* < 0.05). Furthermore, the serotype played a significant role in acid resistance, particularly under the most challenging condition (pH 2.5). The serotype 1/2c strains showed a significantly higher reduction than the other subtypes, highlighting their increased sensitivity to acidic environments (*p* < 0.05) (Figure 4B). This suggests that the serotype can be a factor in determining the ability of *L. monocytogenes* to survive in acidic foods.

### 3.4. Motility Ability

**Figure 5:**
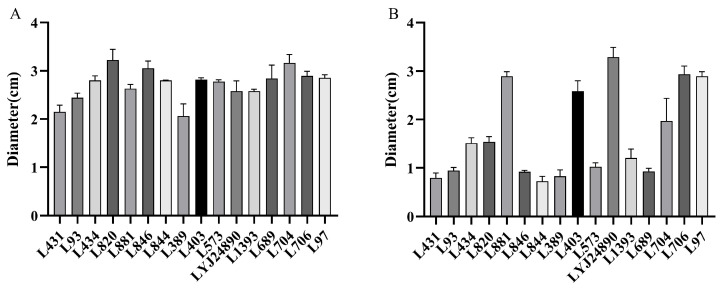
Comparison of swimming (**A**) and swarming (**B**) of *L. monocytogenes*.

Bacterial motility enables cells to move to favorable growth environments or evade unfavorable conditions, and it is related to a strain’s resistance to adverse environments and its virulence. The swimming and swarming abilities of the 16 *L. monocytogenes* strains was measured at 25 °C, and the strains exhibited variability in their swimming and swarming abilities (Figure 5). The swimming diameter of the strains ranged from 2.22 to 3.22 cm, with an average of 2.73 cm. Strains L820 and L704 showed significantly larger swimming diameters than the other strains (*p* < 0.05). The swarming ability displayed even greater variation among the strains, with strain L844 exhibiting the smallest swarming diameter (0.73 cm), and LYJ24890 exhibiting the largest (3.29 cm).

Overall, the lineage I strains displayed larger swimming and swarming diameters than lineage II, indicating stronger motility, although the difference was not significant (Supplementary Figure S3). There were no significant differences in the swimming and swarming abilities among the strains of different serotypes. Although the average swimming diameter was highest for serotype 4b (2.94 cm) and lowest for serotype 1/2c (2.55 cm), these differences were not statistically significant (Supplementary Figure S4). Similarly, while the largest swarming diameter belonged to serotype 1/2c (2.25 cm) and the smallest belonged to serotype 1/2a (1.13 cm), these variations did not reach statistical significance (Supplementary Figure S4). It is noteworthy that the reference strain (ATCC 19112) exhibited swimming and swarming diameters of 2.36 cm and 1.27 cm, respectively, both below the average values observed for the 16 isolated strains (Supplementary Figure S5). This suggests that the isolated strains possess greater overall motility compared to the reference strain.

### 3.5. Adhesion and Invasion of Caco-2 Cells

**Figure 6:**
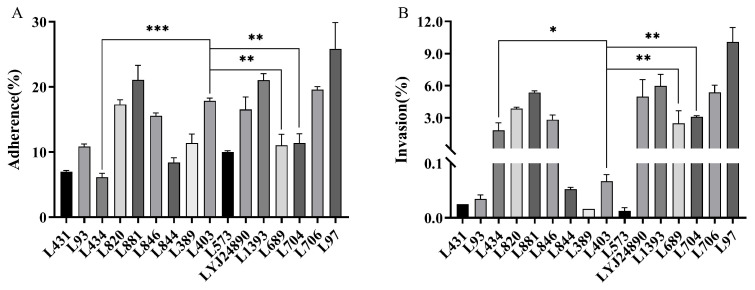
The adhesion (**A**) and invasion (**B**) rates of *L. monocytogenes* to Caco-2 cells. The symbol (*), (**) and (***) indicates significant difference in the representative data (*p* < 0.05), (*p* < 0.01) and (*p* < 0.001).

The ability of *L. monocytogenes* strains to adhere to and invade Caco-2 cells, a widely used in vitro model for studying human intestinal cell function, varies. The average adhesion rate of strains to Caco-2 cells was 14.44%, with a maximum of 25.84% (L97) and a minimum of 6.14% (L434) (Figure 6). The average invasion rate was 2.88%, with a maximum of 10.08% (L97) and a minimum of 0.01% (L573). Strain L97 exhibited significantly higher adhesion and invasion capabilities (*p* < 0.05). Interestingly, some strains exhibited discrepancies between their adhesion and invasion capabilities. For example, while L403 had a significantly higher adhesion rate than strains L434, L689, and L704 (*p* < 0.05), its invasion rate was considerably lower than these strains (*p* < 0.05).

**Figure 7:**
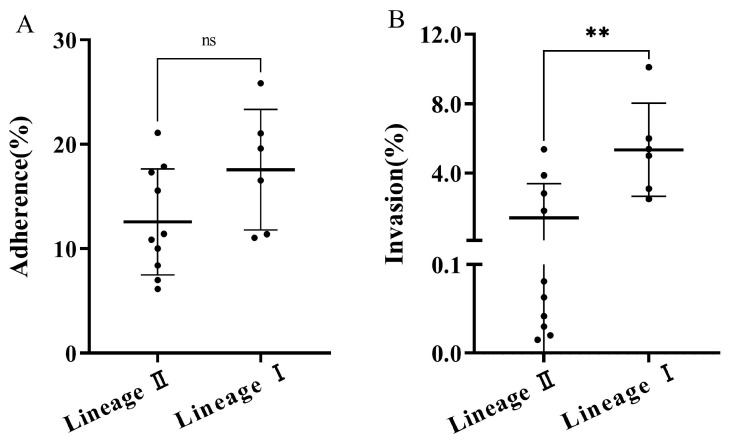
The adhesion (**A**) and invasion (**B**) rates (●) of *L. monocytogenes* strains of the lineages. ns indicates not significant between groups. The symbol (**) indicates significant difference in the representative data (*p* < 0.01).

**Figure 8:**
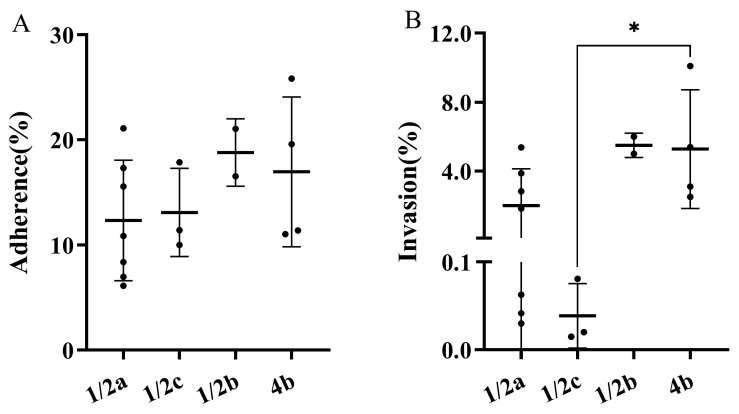
The adhesion (**A**) and invasion (**B**) rates (●) of *L. monocytogenes* strains of the serogroups. The symbol (*) indicates significant difference in the representative data (*p* < 0.05).

Lineage I strains tended to have higher adhesion rates (17.56%) compared to lineage II strains (12.56%), although this difference was not statistically significant (Figure 7A). However, lineage I strains exhibited a significantly higher average invasion rate (5.34%) compared to lineage II strains (1.41%) (*p* < 0.05) (Figure 7B). Serotypes 1/2b and 4b demonstrated higher average adhesion and invasion rates than serotypes 1/2a and 1/2c. However, only the invasion rate of the serotype 4b strains was significantly higher than that of the serotype 1/2c strains (Figure 8). The isolated *L. monocytogenes* strains displayed higher average adhesion and invasion rates compared to the reference strain ATCC 19112 (adhesion rate: 14.01%, invasion rate: 2.31%). However, it is noteworthy that 50% of the isolated strains had lower adhesion rates and 43.75% had lower invasion rates than the reference strain (Supplementary Figure S6).

### 3.6. Survival of Infected Galleria mellonella

**Figure 9:**
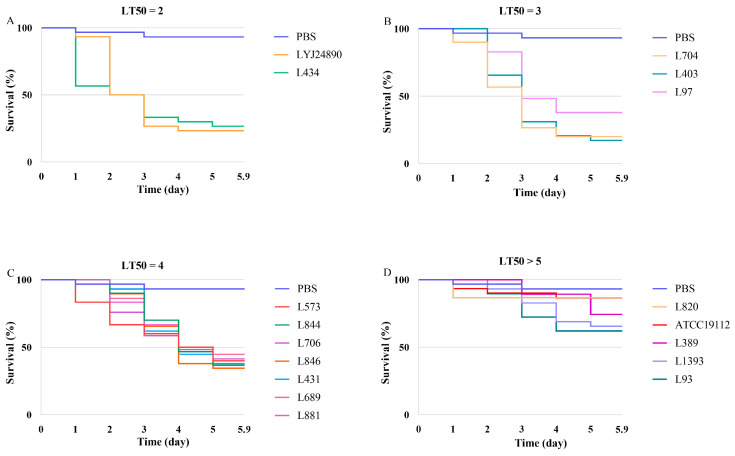
Virulence levels of *L. monocytogenes* isolates in *G. mellonella*. Isolates are grouped by the post-infection incubation time (in days) required to observe 50% or more death of larvae (LT50). (**A**–**C**) indicat isolates with a LT50 of 1 to 4 days, and panel (**D**) shows isolates that did not reach 50% mortality on day 5 postinfection (LT50 > 5).

Insects have immune systems similar to mammals, making insect infection models a rapid, cost-effective, and reliable means to assess the pathogenicity of bacteria. The virulence levels of *L. monocytogenes* strains were evaluated using the *Galleria mellonella* infection model. Over a 5-day observation period, the mortality rate of larvae injected with PBS (negative control) did not exceed 6%. Larvae infected with the isolated strains were continuously monitored for 5 days, and the lethal time of 50% (LT50) was counted in days. The results show that all tested strains were capable of killing the larvae, with LT50 values ranging from 2 to greater than 5 days (Figure 9). Two strains (LYJ24890 and L434) demonstrated the highest virulence, reaching an LT50 of 2 days. Conversely, five strains failed to achieve a 50% mortality rate even after 5 days of infection, indicating lower virulence. Notably, the majority of strains exhibited an LT50 of 4 days (7 out of 16 strains).

The analysis based on lineage revealed that the lineage I strains had a higher average mortality rate after 5 days (60%) compared to the lineage II strains (53%), although this difference was not statistically significant (Supplementary Figure S7). Only one lineage I strain (L1393) had an LT50 exceeding 5 days, representing 1/6 of the tested strains. In contrast, 30% of the lineage II strains did not reach a 50% mortality rate by the end of the observation period. Regarding serotype, the serotype 4b strains exhibited the highest average mortality rate of 62.50%, whereas the serotype 1/2a strains had the lowest average mortality rate at 52.38%. However, no statistically significant differences were observed in the mortality rates among the strains of different serotypes (Supplementary Figure S7). The reference strain, ATCC 19112, exhibited a mortality rate of 13.33% after 5 days of larval infection, significantly lower than the average rate observed for the 16 isolated strains (55.63%). Notably, all strains except L820 displayed higher mortality rates than the reference strain (Supplementary Figure S8).

## 4. Discussion

*L. monocytogenes* have emerged as prominent important foodborne pathogens that are prevalent worldwide. Nearly all reported listeriosis sporadic cases and outbreaks have been transmitted via contaminated food [25]. Because the population behavior of *L. monocytogenes* is heterogeneous, it is important to study a possible correlation between subtypes and their potential pathogenicity. The strains with increased ability for growth, stress resistance, and virulence may lead to increased risk of infection. To decipher the correlation between variable distribution and the pathogenicity of *L. monocytogenes* subtypes, our study evaluated the stress resistance and virulence phenotypes of 16 strains. However, due to the limited number of samples, there were no obvious rules in some indicators among the different subtypes of strains, so the results of our study should be interpreted carefully. In addition, the sample size can be increased for a more in-depth study on the potential pathogenicity of *L. monocytogenes*. By understanding the variability among strains and analyzing the pathogenicity of different subtypes of strains, it is helpful to improve risk assessment and develop more informed risk management.

Our study’s findings on the maximum specific growth rate and lag time of the 16 *L. monocytogenes* strains are consistent with several previous studies [26,27,28,29]. Generally, when compared to the lineage I strains of *L. monocytogenes*, the lineage II strains show a growth and survival advantage under some unfavorable conditions [10,30]. In contrast, we found that the lineage I strains had a higher growth rate compared to the lineage II strains at three growth temperatures, and the difference was significant at 37 °C, which may be the ideal environmental conditions. Muchaamba et al. also observed more rapid growth of lineage I compared to lineage II strains in brain heart infusion broth [17]. Similarly, our results show that the maximum specific growth rate of the serotype 4b isolates was significantly higher than the other subtypes. Unlike previous studies [31,32], strains of serogroup 1/2 may have a growth advantage over serotype 4b. Some authors found that the lag time tends to show differences at a lower growth temperature, and the variation among strains decreases as the temperature reaches the optimal growth temperature [33,34]. The isolates of the food processing environment or food origin predominately belong to serogroup IIa or IIb of lineage II, because they contain more stress tolerance genes, such as acid stress tolerance, cold stress, and biofilm genes, but lineage I isolates have more virulence genes than lineage II isolates [35]. Moreover, our results show that two serotype 4b strains of lineage I grew faster, which should be paid more attention.

The *D*-values obtained in this study are comparable to those reported by Smelt and Brul (2014) and Aryani et al. (2015b) [36,37]. Our results highlight significant variations in heat resistance among the studied strains, with the *D*-values differing by nearly six-fold at the same temperature. Although the differences in heat resistance can be attributed to various external factors, including bacterial age, growth conditions, prior stress exposure, and food composition [36], under the same experimental conditions, this can only be attributed to genetic differences. While no significant differences were observed in the *D*-values between the lineage I and lineage II strains at the tested temperatures, an interesting trend emerged; the *D*-values of the lineage II strains gradually increased with higher temperatures, exceeding those of the lineage I strains. This finding aligns with the observation by De Jesús and Whiting (2003) and suggests a potential heat tolerance advantage for lineage II strains under more challenging conditions [34]. Remarkably, the analysis revealed substantial variations in heat resistance within serotypes. Strains L820 (serotype 1/2a) and L1393 (serotype 1/2b) exhibited significantly higher *D*-values compared to the other strains within their respective serotypes. This observation, supported by findings from Shen et al. (2014), who found similar variability within serotype 1/2a, underscores the importance of strain-specific analysis rather than relying solely on the serotype or lineage for predicting heat tolerance [38]. Heat shock proteins play a crucial role in *L. monocytogenes*’ response to heat treatment [39]. It has been shown that three classes of genes are associated with heat shock, among which class II genes are positively regulated by SigB, and the other two genes are under the negative control of HrcA and CtsR [40,41,42].

Our study demonstrates that under normal acidic conditions (pH 3.5 and 4.5), most isolates exhibited similar acid resistance. However, when exposed to a highly acidic environment (pH 2.5), two strains (L389 and L573) displayed exceptionally high population reduction, mirroring the findings reported by Dykes and Moorhead (2000) [43]. The literature presents conflicting reports on acid resistance variations among lineages. Hingston et al. (2017) observed lower acid susceptibility in lineage I strains [44], while Wu et al. (2022b) found the opposite [45]. Myintzaw et al. (2022), however, reported no significant difference between the lineages [46]. These differences likely stem from the diverse methodological approaches used in different studies. Our results indicate that serotype 1/2a isolates generally exhibited higher acid tolerance compared to the other serotypes, aligning with the findings of Yunge et al. (2020) [47]. Although the observed differences in the population reduction among serotypes under pH 2.5 conditions were not statistically significant, the serotype 1/2a isolates consistently showed the smallest average reduction values. While stress survival islet 1 (SSI-1), containing genes *gadD1* and *gadT1*, is known to contribute to *L. monocytogenes*’ acid tolerance [48,49], the surprising high sensitivity of the two SSI-1-positive strains (L389 and L573) in our study suggests the influence of other genetic elements. This finding, corroborated by Hingston et al. (2017), necessitates further investigation into the complex interplay of various genes and pathways in acid resistance [44]. It is important to note that *L. monocytogenes* employs two additional mechanisms to adapt to low acid stress: the arginine deiminase (ADI) system [44] and F_0_F_1_-ATPase [50]. Additionally, research suggests that weak organic acids are more detrimental to the bacterium compared to strong inorganic acids [51,52].

Motility is associated with virulence as the process is thought to be necessary for *L. monocytogenes* to reach unique niches and persist within the host [53,54]. Our study involved swimming and swarming motility. Although both are mediated by flagella, swimming is a movement in a liquid environment, whereas swarming occurs on a solid surface [55]. Apart from the genetic element of bacteria, environmental factors, such as humidity, temperature, agar thickness, and cell density, also impact motility, highlighting the importance of standardization in motility assays [56]. Our study suggests a trend of stronger motility among the lineage I isolates compared to the lineage II isolates, particularly in swarming. Interestingly, a correlation was observed between strong swarming motility and high invasion rates in strains L434, L820, and L881. This finding aligns with the observations of Dons et al. (2004), who linked swarming motility to enhanced host cell invasion [57].

The ability of food-borne pathogen to adhere to, invade, and survive within host cells is critical for establishing systemic infections. Evaluation of the virulence of *L. monocytogenes* by using Caco-2 cells and *G. mellonella* is widely used. Our results indicate that the serotypes 4b and 1/2b isolates exhibited higher average adhesion and invasion rates compared to serotypes 1/2a and 1/2c. Notably, the invasion rate of the lineage I strains was significantly higher than that of the lineage II strains. These findings are partially consistent with previous studies. Lee et al. and Martinez et al. reported no significant differences in the LT50 among the lineage and serotype strains using the *G. mellonella* model [24,58]. However, these studies also acknowledged that hypervirulent strains were predominantly found within lineage I. This observation aligns with the suggestion by Maury et al. (2017) that the presence of additional Listeria Pathogenicity Island (LIPI-3 and LIPI-4) genes in lineage I isolates might contribute to increased virulence [8]. LIPI-1 encodes key virulence factors, including *actA*, *hly*, *mpl*, *plcA*, *plcB*, and *prfA* [59], with *hly* and *actA* being particularly important. ActA, a surface protein, facilitates bacterial invasion and movement within host cells [60]. Listeriolysin O (LLO), encoded by *hly* gene, performs two critical functions: it destroys vacuoles to release bacteria into the cytoplasm and regulates vacuolar pH to delay phagosome maturation [61]. PrfA, on the other hand, regulates the expression of the *inlA* and *inlB* genes [62]. InlA binds specifically to human E-cadherin, playing a crucial role in cell invasion and crossing the intestinal barrier [63,64]. InlB interacts with various host cell receptors, mediating entry into different cell types [65,66]. Notably, many lineage II isolates possess premature stop codons in the inlA gene, leading to reduced invasion efficiency [67]. Joyce and Gahan observed significantly increased expression of virulence genes (including *actA*, *mpl*, *plcA*, and *prfA*) in the *G. mellonella* model at an incubation temperature of 37 °C [68]. The highly conserved *llsX* gene located on LIPI-3 encodes listeriolysin S (LLS), enhancing the cytotoxic and hemolytic activity of *L. monocytogenes* [69]. LIPI-4, strongly associated with clinical cases, plays a role in the invasion of the central nervous system and placenta [8]. Our results also support the findings of Jaradat and Bhunia (2003), who observed a poor correlation between adhesion and invasion in some strains [70]. High adhesion rates did not necessarily translate to high invasion rates. Furthermore, some lineage II isolates (such as L881 and L820 in cell infection and L403 in the insect larvae model) exhibited high virulence. These observations highlight the complexity of virulence, with factors beyond LIPI presence contributing to its manifestation. The pPplA peptide (peptide pheromone-encoding lipoprotein A) promotes bacterial escape from host cell vacuoles and may also upregulate *prfA* expression [71]. Ling et al. [72] confirmed that *inlF* contributes to bacterial survival within macrophages and facilitates early-stage colonization. Additionally, the LAP-Hsp60 pathway has been identified as another mechanism facilitating the passage of bacteria through the intestinal epithelium [73].

## 5. Conclusions

In our study, 16 strains of *L. monocytogenes* isolated from imported meat revealed distinct differences in growth ability, stress resistance, and virulence among subtypes. Lineage II strains were better able to survive in adverse environments compared to lineage I strains. Specifically, *D*-values of lineage II strains gradually exceeded those of lineage I strains at higher temperatures, and serotype 1/2a isolates generally demonstrated lower acid susceptibility compared to other serotypes. Regarding virulence, lineage I strains showed higher virulence than those of lineage II strains. Significant variations in the growth characteristics, stress resistance, and virulence were observed among different strains, with some lineage I strains displaying high stress resistance and certain lineage II strains exhibiting heightened virulence. The findings from this research can provide valuable insights for the development of effective strategies to mitigate the risks associated with *L. monocytogenes* contamination in the global food supply chain. However, due to the limited number of strains, the correlation between subtype and potential pathogenicity still needs to be explored. Moreover, further investigation is needed to reveal the mechanisms responsible for the correlation.

## Figures and Tables

**Table 1:** *L. monocytogenes* strains used.

	Sources	Lineage	Country	ST ^1^	Serogroup	LIPI ^2^	SSI ^3^
L431	Pork	II	Spain	121	1/2a	LIPI-1 *	SSI-2
L93	Salmon	II	Norway	121	1/2a	LIPI-1 *	SSI-2
L434	Chicken	II	Denmark	155	1/2a	LIPI-1 *	SSI-1
L820	Pork	II	Brazil	155	1/2a	LIPI-1	SSI-1
L881	Pork	II	Netherland	8	1/2a	LIPI-1	SSI-1
L846	Pork	II	Denmark	8	1/2a	LIPI-1	SSI-1
L844	Pork	II	German	8	1/2a	LIPI-1	SSI-1
L389	Pork	II	Denmark	9	1/2c	LIPI-1	SSI-1
L403	Pork	II	Denmark	9	1/2c	LIPI-1 *	SSI-1
L573	Pork	II	Denmark	9	1/2c	LIPI-1	SSI-1
LYJ24890	Beef	I	Uruguay	3	1/2b	LIPI-3, LIPI-1 *	SSI-1
L1393	Beef	I	Australia	3	1/2b	LIPI-3, LIPI-1 *	SSI-1
L689	Pork	I	France	388	4b	LIPI-4, LIPI-1	–
L704	Pork	I	Spain	388	4b	LIPI-4, LIPI-1 *	–
L706	Pork	I	Spain	4	4b	LIPI-3, LIPI-4, LIPI-1 *	–
L97	Pork	I	German	4	4b	LIPI-3, LIPI-4, LIPI-1 *	–

^1^ Sequence typing. ^2^ *Listeria* pathogenicity island. ^3^ Stress survival islets. * Six other virulence genes were present, except *actA*.

## Data Availability

The data are contained within the article.

## Supplementary Materials

The following supporting information can be downloaded at: https://www.mdpi.com/article/10.3390/microorganisms12020345/s1, Figure S1: The *D*-value of *L. monocytogenes* strains at 60 °C (A), 62.5 °C (B), and 65 °C (C); Figure S2: Population reduction of *L. monocytogenes* strains at pH 2.5 (A), pH 3.5 (B), and pH 4.5 (C); Figure S3: Comparison of swimming (A) and swarming (B) of *L. monocytogenes* strains of the lineages; Figure S4: Comparison of swimming (A) and swarming (B) of *L. monocytogenes* strains of the serogroups; Figure S5: Comparison of swimming (A) and swarming (B) of *L. monocytogenes* strains; Figure S6: Comparison of adhesion (A) and invasion (B) of *L. monocytogenes* strains; Figure S7: Comparison of virulence of t *L. monocytogenes* of the lineages (A) and serogroups (B) in *Galleria* model; Figure S8: Comparison of virulence of *L. monocytogenes* in *Galleria model*; Table S1: The growth characteristics parameters *μ_max_* and *λ* of *L. monocytogenes* strains.
