# Supplementary material for: Comparative Analysis of Growth, Survival, and Virulence Characteristics of Listeria monocytogenes Isolated from Imported Meat
Source: Microorganisms. 2024 Feb 7;12(2):345. doi: 10.3390/microorganisms12020345 (PMC10891628; doi:10.3390/microorganisms12020345)
Supplement: Supplementary file 1 [file microorganisms-12-00345-s001.zip › microorganisms-2838451-supplementary.pdf]

microorganisms

MDPI

Article

Comparative Analysis of Growth, Survival, and Virulence Characteristics of *Listeria monocytogenes* Isolated from Imported Meat

Xinye Pan [1,†], Jinling Shen [2,†], Yi Hong [1], Yufan Wu [3], Dehua Guo [2], Lina Zhao [2], Xiangfeng Bu [1], Leijie Ben [1] and Xiang Wang [1,*]

Supplementary Materials

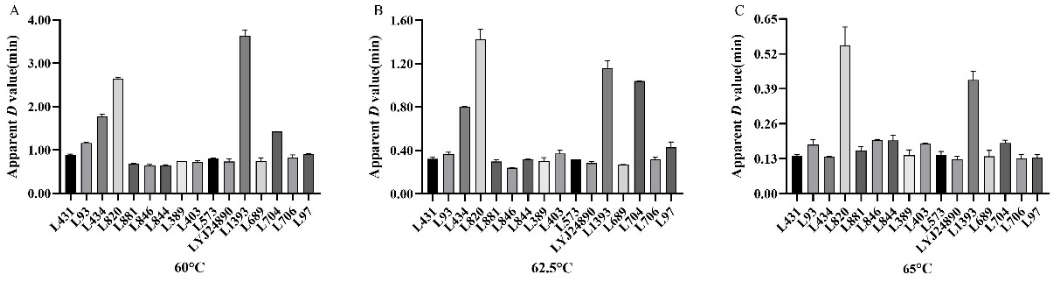

Figure S1. The *D*-value of *L. monocytogenes* strains at 60°C (A), 62.5°C (B), and 65°C (C).

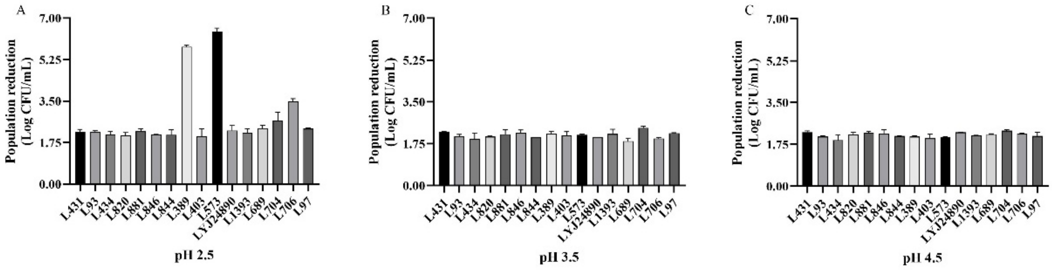

Figure S2. Population reduction of *L. monocytogenes* strains at pH 2.5 (A), pH 3.5 (B), and pH 4.5 (C).

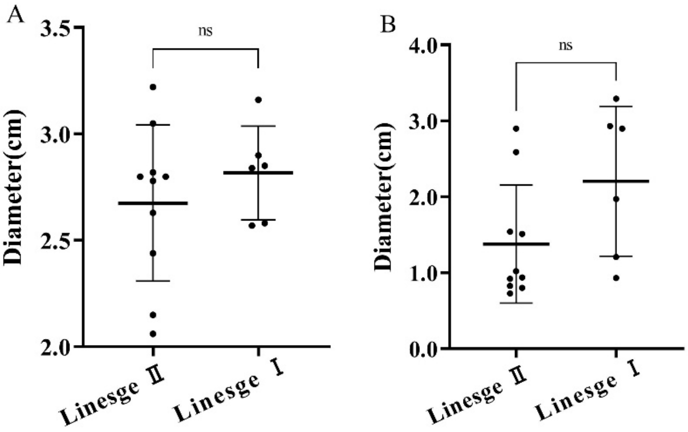

Figure S3. Comparison of swimming (A) and swarming (B) of *L. monocytogenes* strains of the lineage.

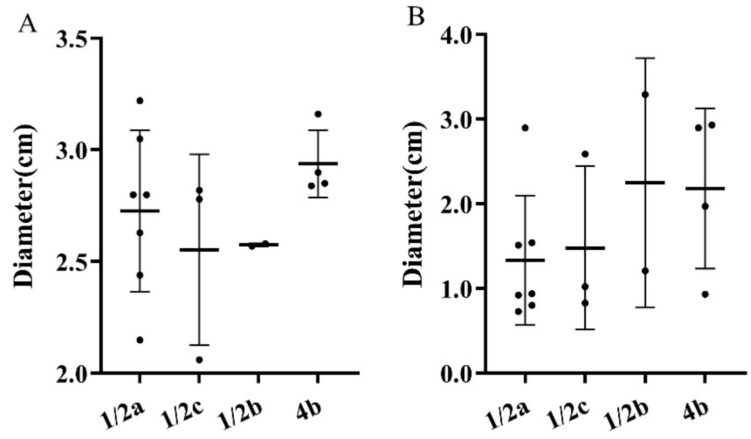

Figure S4. Comparison of swimming (A) and swarming (B) of *L. monocytogenes* strains of the serogroup.

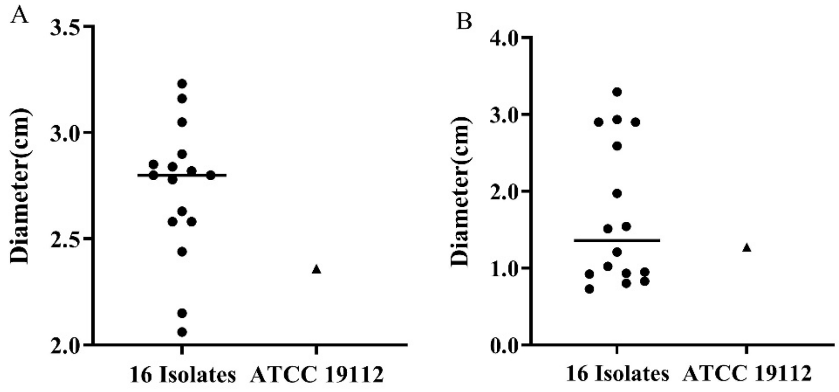

Figure S5. Comparison of swimming (A) and swarming (B) of *L. monocytogenes* strains.

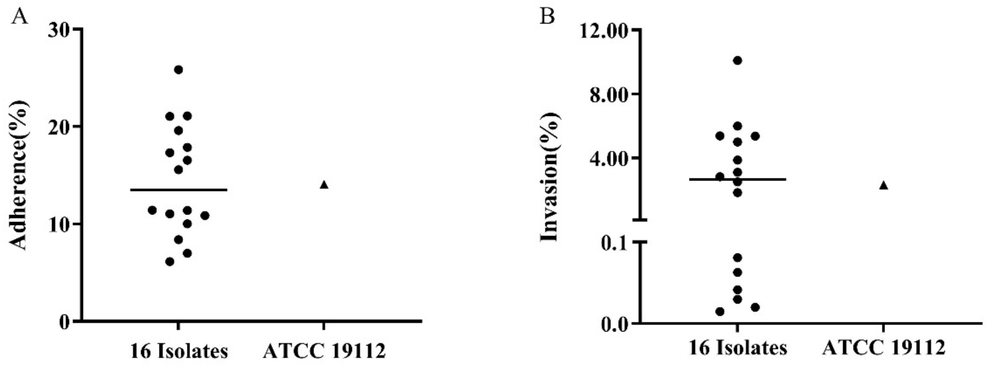

Figure S6. Comparison of adhesion (A) and invasion (B) of *L. monocytogenes* strains.

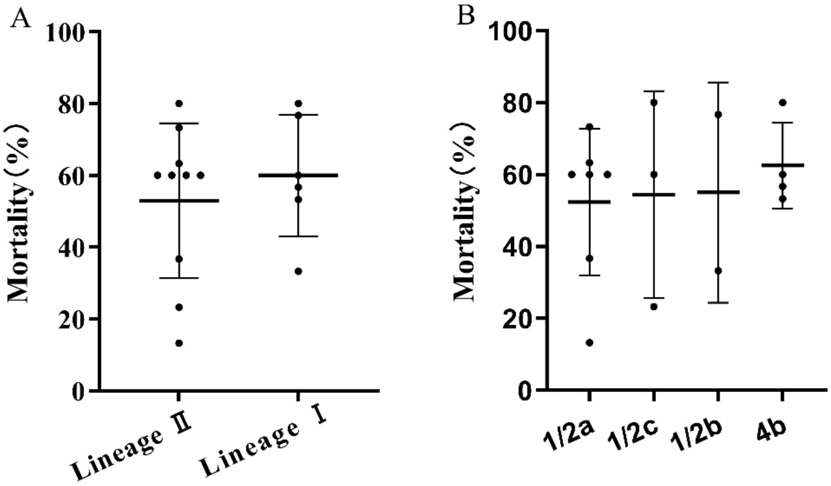

Figure S7. Comparison of virulence of t *L. monocytogenes* of the lineage (A) and serogroup (B) in *Galleria* model.

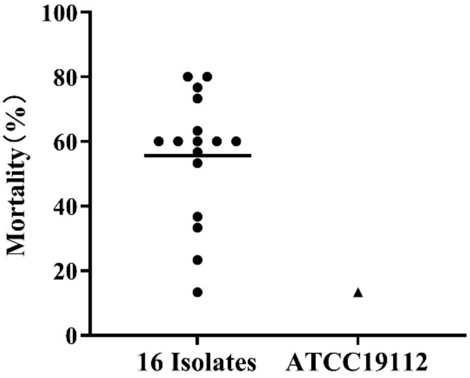

Figure S8. Comparison of virulence of *L. monocytogenes* in *Galleria* model.

Table S1. The growth characteristics parameters μ_{max} and λ of *L. monocytogenes strains*.

Strain	25 °C		30 °C		37 °C	
	μ_{max}(h^{-1})	λ(h)	μ_{max}(h^{-1})	λ(h)	μ_{max}(h^{-1})	λ(h)
L431	0.73 ± 0.01	2.79 ± 0.11	0.92 ± 0.02	2.55 ± 0.20	0.96 ± 0.01	2.00 ± 0.08
L93	0.68 ± 0.00	3.52 ± 0.12	0.90 ± 0.00	2.69 ± 0.04	1.04 ± 0.00	2.34 ± 0.11
L434	0.76 ± 0.00	2.15 ± 0.10	0.94 ± 0.01	2.15 ± 0.18	1.08 ± 0.03	2.23 ± 0.15
L820	0.62 ± 0.01	2.52 ± 0.09	0.93 ± 0.02	2.07 ± 0.23	1.03 ± 0.04	2.13 ± 0.27
L881	0.73 ± 0.00	2.70 ± 0.25	0.94 ± 0.00	2.04 ± 0.11	1.12 ± 0.07	1.65 ± 0.31
L846	0.62 ± 0.00	2.72 ± 0.05	0.93 ± 0.02	2.33 ± 0.16	1.06 ± 0.02	1.86 ± 0.07
L844	0.62 ± 0.01	2.85 ± 0.16	0.95 ± 0.01	2.02 ± 0.06	1.08 ± 0.02	2.32 ± 0.04
L389	0.71 ± 0.00	2.90 ± 0.04	0.92 ± 0.00	2.33 ± 0.20	1.11 ± 0.00	1.75 ± 0.05
L403	0.61 ± 0.00	2.66 ± 0.09	0.93 ± 0.01	2.57 ± 0.04	0.95 ± 0.01	1.72 ± 0.17
L573	0.69 ± 0.01	3.34 ± 0.21	0.94 ± 0.03	1.85 ± 0.08	1.02 ± 0.01	2.23 ± 0.05
LYJ24890	0.70 ± 0.00	3.11 ± 0.01	0.91 ± 0.02	2.16 ± 0.19	1.18 ± 0.00	1.72 ± 0.16
L1393	0.61 ± 0.00	2.39 ± 0.01	0.94 ± 0.00	2.57 ± 0.03	1.08 ± 0.01	1.81 ± 0.07
L689	0.73 ± 0.00	3.06 ± 0.26	0.92 ± 0.01	2.32 ± 0.09	1.13 ± 0.01	2.33 ± 0.09
L704	0.73 ± 0.01	2.87 ± 0.05	0.93 ± 0.00	2.41 ± 0.17	1.07 ± 0.00	1.85 ± 0.10
L706	0.77 ± 0.01	2.21 ± 0.43	0.96 ± 0.01	2.78 ± 0.09	1.20 ± 0.00	1.68 ± 0.29
L97	0.74 ± 0.00	3.55 ± 0.12	0.98 ± 0.01	2.75 ± 0.15	1.24 ± 0.03	1.67 ± 0.17
Avg ± variation	0.69 ± 0.06	2.83 ± 0.42	0.93 ± 0.02	2.35 ± 0.28	1.09 ± 0.08	1.96 ± 0.26
